# Identification of two QTLs, BPH41 and BPH42, and their respective gene candidates for brown planthopper resistance in rice

**DOI:** 10.1038/s41598-022-21973-z

**Published:** 2022-11-02

**Authors:** Han Qi Tan, Sreekanth Palyam, Jagadeesha Gouda, Prakash P. Kumar, Santhosh Kumar Chellian

**Affiliations:** 1 Department of Biological Sciences, National University of Singapore, Singapore, Singapore; 2 Straits Biotech Pte. Ltd., Singapore, Singapore; 3 SeedWorks International Pvt. Ltd., Hyderabad, India

**Keywords:** Plant breeding, Plant biotechnology, Agricultural genetics

## Abstract

The brown planthopper (BPH) is the leading cause of insect damage to rice plants and BPH infestations have caused profound losses in rice production since the 1970’s. There is an urgent need to discover new BPH resistance genes to ensure the successful production of rice. Here, a new BPH resistance source provided by SeedWorks International Pvt. Ltd., SWD10, was used for this purpose. QTL mapping using 232 F_2_ progenies and 216 polymorphic markers revealed two dominant BPH resistance QTLs, BPH41 and BPH42, located on chromosome 4. BPH resistance mechanism test revealed that antibiosis and antixenosis mechanisms both play a role in BPH resistance conferred by these two QTLs. The QTLs were delimited between markers SWRm_01617 and SWRm_01522 for BPH41, and SWRm_01695 and SWRm_00328 for BPH42. Additionally, using RNA-seq data of lines containing the resistant QTLs, we shortlisted four and three gene candidates for BPH41 and BPH42, respectively. Differential gene expression analysis of lines containing the QTLs suggested that SWD10 BPH resistance is contributed by the plant’s innate immunity and the candidate genes may be part of the rice innate immunity pathway. Currently, the newly identified QTLs are being utilized for breeding BPH resistant rice varieties and hybrids.

## Introduction

The BPH is a major pest plaguing all leading rice growing countries. Not only is the BPH directly causing damage to the rice plants by feeding on the phloem sap, it is also a vector that transmits other rice diseases such as grassy stunt virus and rice ragged stunt virus^1^. Economic loss caused by BPH is estimated to be more than 300 million dollars annually^2^. Major BPH outbreaks have been more frequent post Green Revolution, the BPH is named the ghost of the Green Revolution^3^ and is arguably the major pest problem for modern rice growers.

Frequent BPH outbreaks can be related to the interplay between three major factors, which are: initial population, ecosystem vulnerability and stochastic weather conditions^4^. In addition, modern agricultural practices such as heavy use of nitrogen fertilizers in rice farming exacerbates the problem by improving plant nutritional condition for herbivores, and reducing host plant resistance^5^. More recently, BPH nymphs were also observed to mature faster and gravid adult female BPH were found to lay more eggs in modern agricultural conditions^6,7^.

Another facet of modern agriculture is the use of crop protection chemicals. Crop protection chemicals are the most commonly used method to address insect infestation. In 2011, India used a total of 36,100 tons of insecticides to control the damage from insects, this corresponds to almost 65% of the total pesticides used in the country based on records^8^. However, specifically for BPH control, insecticides have been reported to be ineffective^9^. In fact, after the first wave of major BPH outbreaks in the 1970s, researchers discovered that the use of insecticides have exacerbated BPH outbreaks, which lead to the introduction of the integrated pest management (IPM) strategy. However, the IPM strategy was unable to control BPH outbreaks due to lack of consistent government intervention and difficulty to implement across large areas over long periods of time^10^. Thereafter, farmers have consistently relied on using crop protection chemicals for BPH control leading to another wave of major BPH outbreaks in the early 2000s^11,12^.

The ineffective control of BPH due to the factors above lead to a growing awareness for a need to develop resistant/tolerant genotypes against BPH. The logical approach to BPH control would be to use host-plant resistance. Therefore, identification and deployment of new genes for BPH resistance in modern high yielding genotypes is an important strategy to reduce the pest damage.

Currently, approximately 40 BPH resistance QTLs or genes have been identified. Many of these QTLs or genes are concentrated on chromosomes 4, 6 and 12. However, only 11 BPH resistant genes were cloned. These cloned QTLs are BPH1/9, BPH3, BPH6, BPH14, BPH18, BPH29 and BPH32. Three cloned genes, BPH1/9, BPH18 and BPH32, are located on chromosome 12, two cloned genes, BPH6 and BPH3, are located on chromosome 4, and one cloned gene each on chromosomes 6 and 3, viz., BPH29 and BPH14, respectively^1,13–19^.

Focusing on chromosome 4, at least nine QTLs and two genes contributing to BPH resistance have been identified. There are two major QTL clusters on the short arm of chromosome 4. The first QTL cluster is located near the telomere end of chromosome 4. It contains two QTLs, BPH30 and BPH33^20,21^. The second cluster is located closer to the centromere on the short arm of chromosome 4. This cluster contains one cloned gene, BPH3, and five QTLs, BPH12, BPH15, BPH17, BPH20(t) and BPH22(t)^14,22–26^. On the long arm of chromosome 4, there is one major QTL cluster identified for BPH resistance. This cluster contains one cloned gene, BPH6, and two QTLs, BPH27(t) and BPH34^19,27,28^. Based on the number of QTLs and genes that were identified for BPH resistance on chromosome 4, only two genes were cloned on chromosome 4 despite the many works to identify and map new sources of QTLs for BPH resistance.

The large number of mapping studies attempting to identify new QTLs and or genes were driven by the need to identify new BPH resistant sources. This is because resistance conferred by some BPH resistance genes and/or QTLs have started to break down due to high selection pressure. For example, the resistance conferred by the first discovered BPH QTLs, BPH1 and BPH2 were reported to have broken down in many Asian countries as early as the 1970s^29^. Limited BPH resistance sources due to specific interaction between BPH resistance QTLs with BPH biotypes coupled with the breaking down of resistance provided by BPH resistance genes/QTLs, have reduced breeders’ selection to a limited set of deployable BPH resistance genes/QTLs to improve BPH resistance in rice.

Furthermore, there is limited understanding of the molecular mechanisms of action of the cloned BPH resistance genes. The understanding of the plant innate immunity and the roles of the BPH resistance genes identified have been summarized into two major categories, the effector-triggered immunity (ETI) and the pathogen-associated molecular pattern (PAMP)-triggered immunity (PTI)^30^. Proteins containing leucine rich repeat domain (LRR) domains and lectin kinases have been identified as part of the innate immunity in other species and have been well documented to play a major role in activating the plant ETI and/or PTI^31^.

The interplay between ETI and PTI was suggested by the zig-zag-zig model where PTI is initially triggered upon the plant recognizing PAMP, triggering an immune response from the plant. The pathogen then releases effectors that attempts to block the plant immune response. Resistant plants will be able to detect these effectors and enhance their immunity through ETI. Resistance of the plant depends highly on recognizing pathogen effectors^32^. More recently, it has been elucidated that PTI and ETI reciprocally enhance each other. When PTI is induced, ETI gets activated, which, in turn, enhances several key responses of PTI, such as increased production of reactive oxygen species (ROS), increased callose deposition and an increased transcription of signaling components such as MAPK and MPK signaling kinases. Also, when PTI is activated, PTI will enhance the response of ETI by inducing hypersensitive response towards the pathogens^33^.

To date, a total of nine genes have been functionally characterized for BPH resistance and they were found to be involved in the plant innate immunity pathway^34^. Two genes, *BPH3* and *BPH15*, encode plasma membrane localized lectin receptor kinases, while six genes, *BPH9*, *BPH14*, *BPH18*, *BPH26*, *BPH29*, and *BPH32*, encode LRR proteins^35^, *BPH6* is the only gene that encodes a protein that localizes to the exocyst^19^. *BPH6* was found to enhance exocytosis and strengthen the cell wall, therefore, improving BPH resistance^19^. However, the effectors or PAMPs that trigger ETI and PTI within the BPH resistant plants are yet to be identified.

It is evident that plant innate immunity plays a major role in conferring disease resistance. R genes, such as those encoding for LRR proteins, are robust and often provide strong immunity^36^. Since many of the resistance genes identified in the BPH resistance QTLs are part of the plant innate immunity system, it is imperative to discover more sources of BPH QTLs or genes for different BPH populations. This can help to improve BPH resistance in rice and to ensure food security and farmer income in the future.

In this study, we attempted to identify a new BPH resistance source using lines supplied by SeedWorks International Pvt. Ltd. The aim of this study is broken down into four parts: (1) to identify and delimit the QTLs that contribute to BPH resistance from the rice line SWD10, (2) to identify potential gene candidates of the QTLs, (3) to understand the BPH resistance mechanisms contributed by the QTLs and (4) to determine if SWD10 is a new source of BPH resistance.

## Results

### Identification of new BPH resistant QTLs

An F_2_ population consisting of 232 individuals was generated by crossing the resistant donor, SWD10, with a susceptible parent, SWR66. To ensure the results from this study are relevant to the rice breeding programs in India, the F_2_ population was screened with BPH population collected from the rice fields of Rajahmundry, India, a BPH hotspot. The Indian BPH biotype is generally classified as biotype 4. The average resistance scores of the F_3_ progenies derived from individual F_2_ lines were used for each F_2_ individual. A skewed normal distribution of the average resistance scores was observed (Figure S1). There were more susceptible F_2_:F_3_ genotypes compared to resistant phenotypes. Also, there were no genotypes that were as resistant as SWD10. Based on the skewed distribution of the resistance scores and number of resistant lines observed, we postulate that there are more than one QTL contributing to BPH resistance from the SWD10 resistant line.

**Figure 1:**
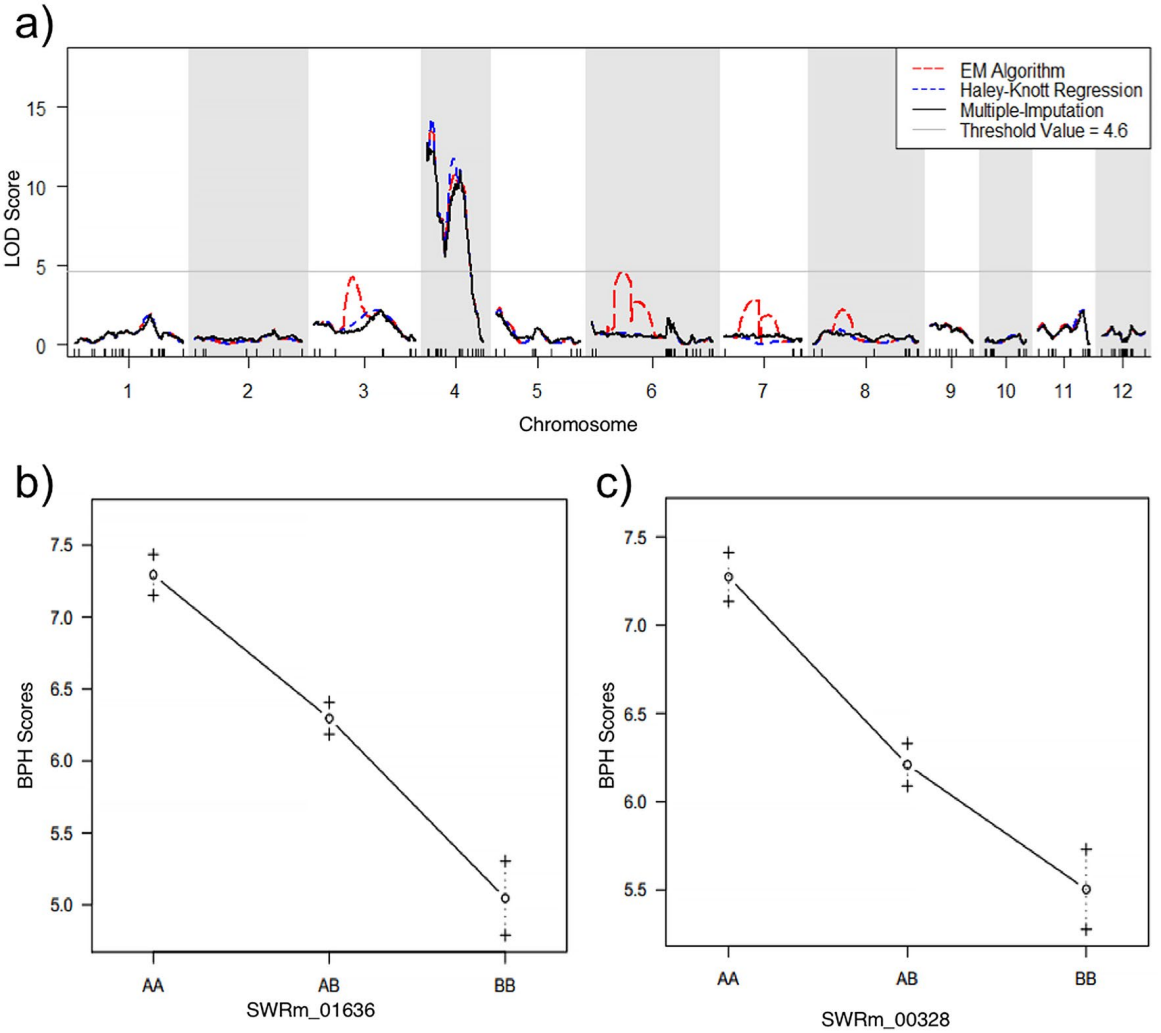
QTL mapping and effect plots of SWR66 × SWD10 F_2_ mapping population. (**a**) QTL mapping results of F_2_ mapping population. Results of three algorithms used are indicated with red (EM algorithm), blue (Haley Knott Regression) and black (multiple imputation) lines. The grey line indicates the LOD threshold value of 4.6 detected by performing a 1000 permutation test using the EM algorithm. The LOD threshold values detected by 1000 permutation tests for the HK algorithm and multiple imputation are 4.34 and 4.19, respectively. Two major QTLs are identified on chromosome 4. The highest peak is designated as BPH41 (LOD = 12.30), and the second peak is designated as BPH42 (LOD = 11.08). (**b**,**c**) Effect plots of markers SWRm_01636 and SWRm_00328. These markers are located closest to the peak for BPH41 and BPH42, respectively. AA, AB and BB represent homozygous susceptible, heterozygous and homozygous resistant genotype, respectively. The BPH resistance scores on the y-axis are the mean resistance score for each genotype (mean ± s.e; n = 232).

QTL mapping was carried out using the average resistance scores of 232 F_2_ individuals, and their respective genotypes were generated using 216 polymorphic single nucleotide polymorphic (SNP) markers spanning across all 12 chromosomes of rice. Three separate QTL mapping algorithms: expectation maximization algorithm (EM), Haley Knott regression (HK) and multiple imputation method (imp) were used for this purpose. Based on the 1000 permutation test, the threshold value of 4.6 was used as it was the highest threshold value among all three algorithms. All three algorithms detected two QTLs (BPH41 and BPH42) located on chromosome 4 with maximum LOD values of 12.30 and 11.08, respectively. These QTLs were named as BPH41 and BPH42 (Fig. 1a). The sizes of the QTLs straddling the QTL peaks are approximately 6.0 million bp and 6.3 million bp, and the markers closest to the QTL peaks are SWRm_01636 and SWRm_00328 for BPH41 and BPH42, respectively.

An effect plot was generated for the markers closest to the peak for each QTL to understand the contribution of each QTL on BPH resistance. Using the marker closest to the QTL peak, the presence of a homozygous SWD10 genotype, indicated as BB, for the marker SWRm_01636 increases BPH resistance of the plant from a susceptible score of 7.3 to a moderate resistance score of 5.3 (Fig. 1b). A homozygous SWD10 genotype at marker SWRm_00328 behaves similarly to SWRm_01636 by increasing BPH resistance score from 7.3 to 5.5 (Fig. 1c). The contributions to BPH resistance by BPH41 and BPH42 are 23.6% and 20.6%, respectively (Table 1).

**Table 1:** QTLs detected for SWR66 × SWD10 F_2_ mapping population.

	LOD	%var	p value (F)
BPH41	12.30	23.596	9.99e−15***
BPH42	11.08	20.591	5.68e−13***

Significant codes: 0 '***' 0.001 '**' 0.01 '*' 0.05 '.' 0.1 ' ' 1.

### Delimiting QTL size of BPH41 and BPH42

BPH41 and BPH42 are both located on chromosome 4 and are genetically linked. The genetic distance between the closest markers delimiting the QTL regions for BPH41 and BPH42 is 17.56 cM (Data S1). To further verify and delimit the QTLs, recombinant lines containing only BPH41 or BPH42 were generated. A total of 14,368 plants from advanced generations of F_3_ to F_6_, BC_1_F_1_ to BC_1_F_4_ and BC_2_F_1_ to BC_2_F_4_ were screened using SNP markers to separate the two QTLs and to generate recombinant lines for each QTL.

**Figure 2:**
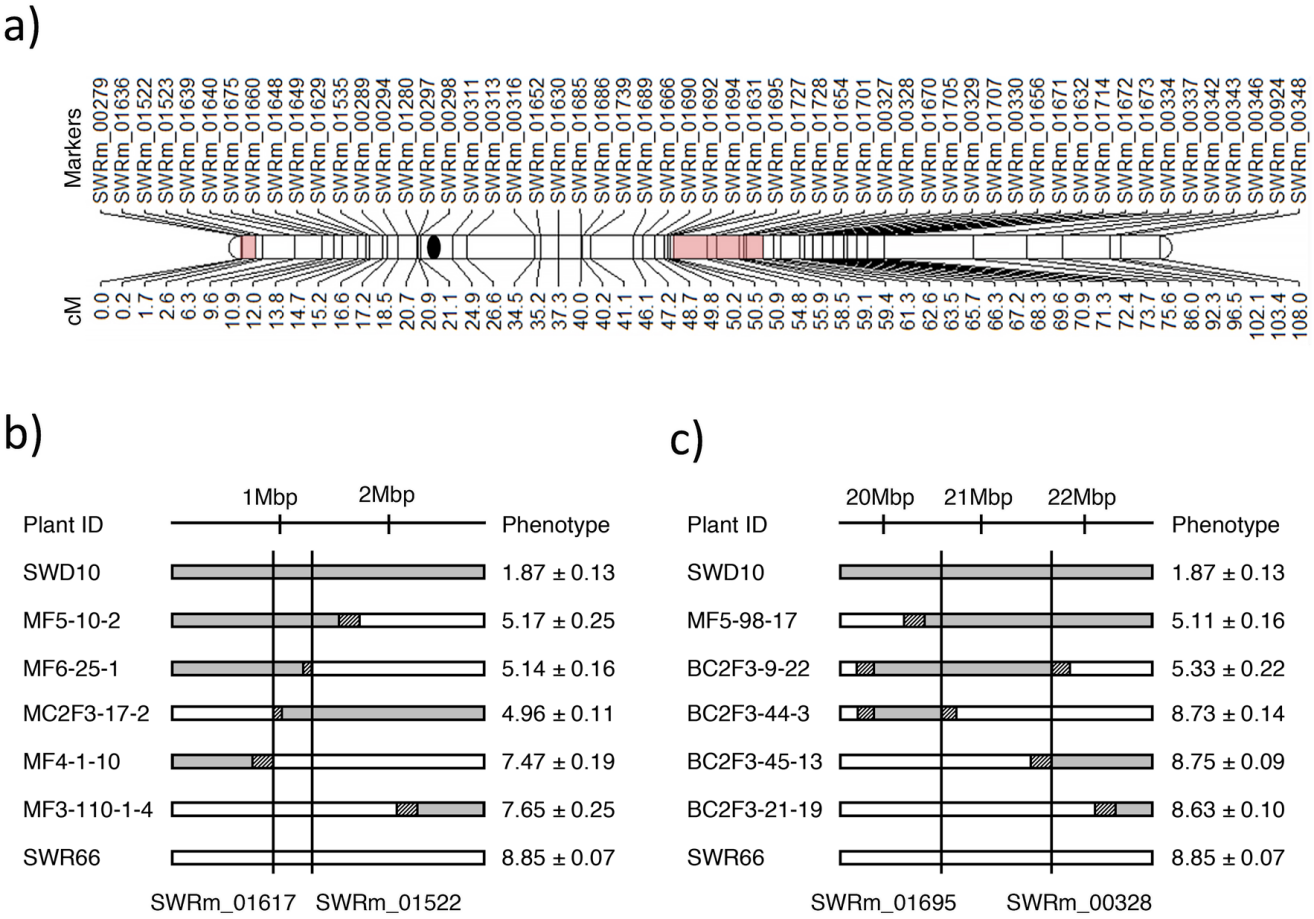
Delimiting BPH41 and BPH42. (**a**) Overview of polymorphic markers and their respective genetic distance on chromosome 4. The delimited QTLs for BPH41 and BPH42 are indicated with red boxes. (**b**,**c**) key recombinant lines used for delimiting BPH41 and BPH42. Six recombinant lines delimiting each QTL are illustrated. The average resistance scores for each recombinant lines and the parents are indicated on the right of each genotype (mean ± s.e; n = 60). Solid boxes reflect the homozygous resistant genotype, open boxes reflect homozygous susceptible genotype and striped boxes reflect unknown genotype.

Concurrently, whole genome resequencing was conducted on both SWR66 and SWD10 to identify polymorphic SNP markers between the parents. The polymorphic SNPs saturating both QTL regions were converted into KASPar assays to genotype the advanced generation lines (Fig. 2a).

BPH phenotyping was conducted on 249 selected recombinant lines from the advanced generations. Recombinant individuals generated from lineages giving consistent resistance scores across multiple generations were used to delimit the QTL regions. An average resistance score of less than 5.5 was used as a cutoff for resistant lines (Fig. 5a). The cutoff was based on the results of the effect plots of from the QTL mapping of BPH41 and BPH42 and on the average resistance scores observed in the initial QTL mapping population. The average resistance scores were 5.28 and 5.59 for lines containing resistant alleles of BPH41 and BPH42, respectively. To determine the susceptible lines, the cutoff score of more than 7.0 was used. This was decided based on the standard scoring method published from IRRI^37,38^.

Results indicate that the QTLs were located between markers SWRm_01617 and SWRm_01522 for BPH41, and SWRm_01695 and SWRm_00328 for BPH42 (Fig. 2b,c). The QTLs of BPH41 and BPH42 were refined from 6.0 million base pairs to 0.54 million base pairs and 6.3 million base pairs to 1.28 million base pairs, respectively. Furthermore, genetic locations of both delimited QTLs were compared to the R498 genome in an attempt to identify potential gene candidates. There are 47 and 162 annotated genes within the delimited regions of BPH41 and BPH42, respectively (Data S2, S3).

Despite screening many plants to delimit the QTLs, the delimited region remained fairly large and we were not able to further fine map the QTLs. There were also numerous genes within each QTL, therefore, we employed a different method to further shortlist potential gene candidates conferring BPH resistance.

### Identification of potential gene candidates

We employed an RNA-seq approach to further narrow down candidate genes for each QTL. A 2-week-old susceptible and resistant parent along with BC_2_F_2_ lines containing either the homozygous resistant allele of BPH41 or BPH42 in the susceptible parent background were used to identify differentially expressed genes (DEGs) related to BPH resistance.

**Figure 3:**
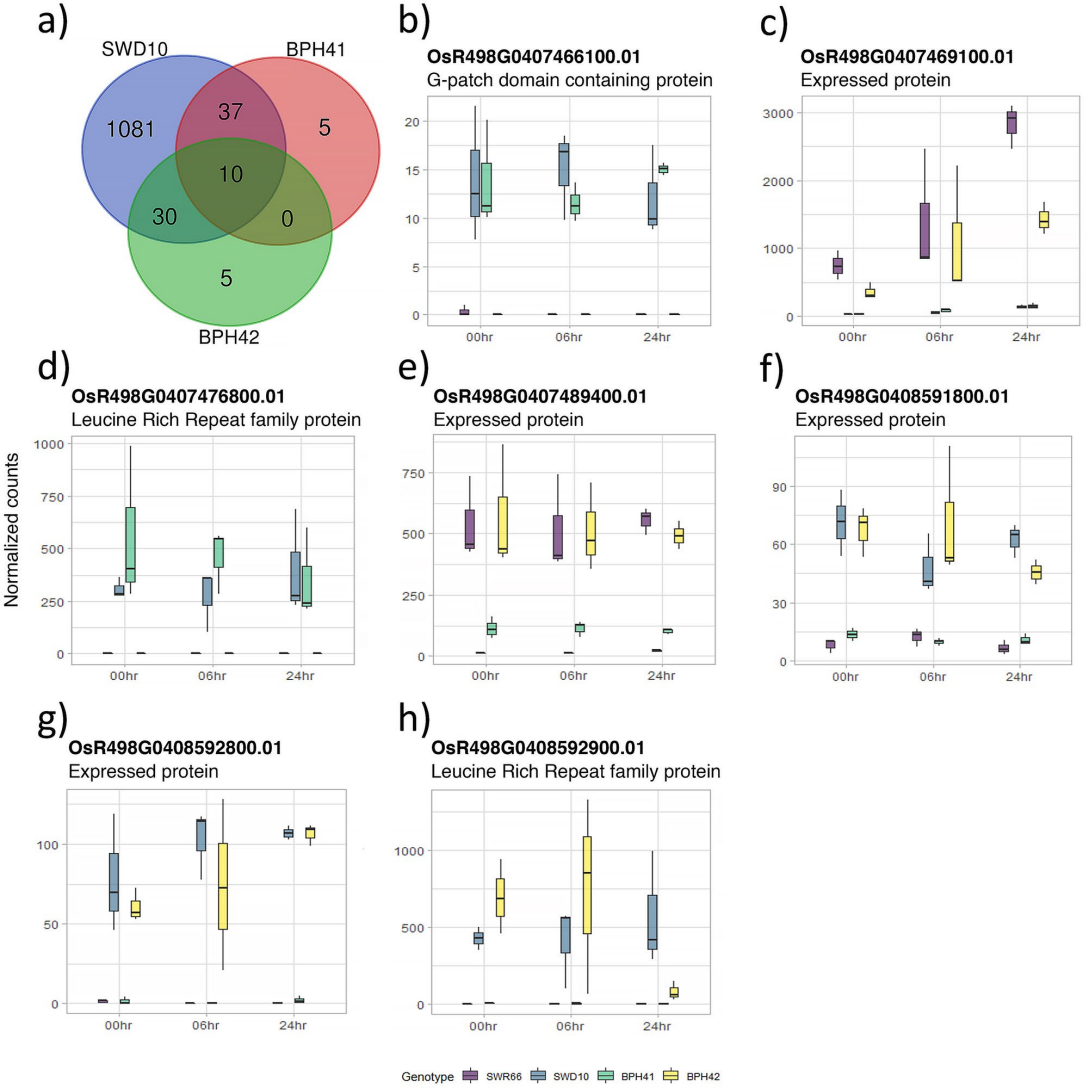
Venn diagram of genotypes against SWR66 at 0 h and boxplots of potential gene candidates for BPH41 and BPH42. All genotypes are lines containing the respective resistant allele of the indicated QTLs in the SWR66 background except parental and control lines. (**a**) Venn diagram illustrating the number of differentially expressed genes compared to SWR66 for SWD10, BPH41 and BPH42 genotypes at 0 h. The cut-off values used to identify DEGs of interest are, p value < 0.001 and absolute log_2_ fold change > 2. (**b**–**h**) boxplot of normalized count values for seven shortlisted genes in BPH41 and BPH42 QTLs. Normalized gene counts for three timepoints, 0 h, 6 h and 24 h and four genotypes are illustrated. Boxplots (**b**–**e**) are gene candidates located within BPH41 and boxplots (**f**–**h**) are gene candidates located within BPH42.

First, we compared the DEGs between SWD10 and SWR66 at 0 h after BPH release. A total of 1158 DEGs between SWR66 and SWD10 (Fig. 3a) were identified. A GO term and KEGG pathway analysis on the 1158 DEGs identified only a handful of biological process (Table 2). The lack of statistically significant GO terms and KEGG pathways reflected the low number of functionally annotated genes from the pool of 1158 DEGs in SWD10. There were many DEGs in SWD10 when compared to SWR66 before BPH infestation, this was the first indication that when challenged with BPH SWD10 responds differently from SWR66.

**Table 2:** Enriched GO terms and KEGG pathways between SWD10 and SWR66 at 0 h.

	**Term**	**SWD10**
**GO.ID**		
GO:0006468	Protein phosphorylation	3.50E−12
GO:0009772	Photosynthetic electron transport in photosystem II	3.00E−03
GO:0006974	Cellular response to DNA damage stimulus	2.20E−02
GO:0010112	Regulation of systemic acquired resistance	2.30E−02
GO:0031146	SCF-dependent proteasomal ubiquitin-dependent protein catabolic process	2.70E−02
GO:0015986	ATP synthesis coupled proton transport	3.90E−02
GO:0000910	Cytokinesis	3.90E−02
**KEGG.ID**		
osa01212	Fatty acid metabolism	4.64E−02

To further understand the differences between the responses of SWD10 and SWR66 against BPH, we compared the DEGs between both plants at timepoints of 0 h (baseline) with 6 h and 24 h after infestation. Comparing 0 h and 6 h post infestation, only one and two DEGs were identified for SWD10 and SWR66, respectively (Figure S2a). For the timepoints between 0 and 24 h, the number of DEGs identified were six and 414 for SWD10 and SWR66, respectively, with two overlapping genes (Figure S2b). These results suggested that the reaction towards BPH infestation produced a stronger response 24 h post infestation. While SWR66 is more sensitive towards BPH infestation, SWD10 resistance towards BPH seems to be conferred by being insensitive towards BPH infestation. Based on the low number of DEGs identified in SWD10 after BPH infestation, we postulated that resistance towards BPH in this genotype may be due to genes that are already expressed in the resting state of SWD10.

Based on the above inference, we looked at the subset of the DEGs expressed at 0 h between the resistant genotypes to identify potential resistant gene targets for BPH41 and BPH42. A total of 37 DEGs common between BPH41 and SWD10, and 30 DEGs common between BPH42 and SWD10, were of interest (Fig. 3a). Gene expression profiles of the DEGs located within the QTLs were matched against the gene expression profiles of the same genes in SWD10 and SWR66 at 0 h. DEGs that exhibited a similar gene expression profile to SWD10 but with an opposite gene expression profile to SWR66 were shortlisted as potential gene candidates. Based on the above criteria, we narrowed down four and three genes within the QTLs as potential gene candidates for BPH41 and BPH42, respectively (Fig. 3b–h). For each BPH QTL, a leucine rich repeat (LRR) domain containing gene was identified. Gene expression profiles for the candidate genes were verified with qRT-PCR (Figure S3). Five genes reflected the same gene expression levels as the RNA-seq data. The remaining two genes could not be verified due to the failure to design specific primers and the presence of high GC content within the gene sequences.

### BPH resistance mechanisms of BPH41 and BPH42 and dominance test

Antibiosis and antixenosis tests were carried out to determine the resistance mechanisms conferred by the resistant alleles of BPH41 and BPH42. The antibiosis test was conducted to determine if the QTLs confer resistance by mortally affecting the BPH that were feeding on the plant, whereas the antixenosis test was used to determine if the resistance was conferred by deterring the BPH to rest on the plant.

**Figure 4:**
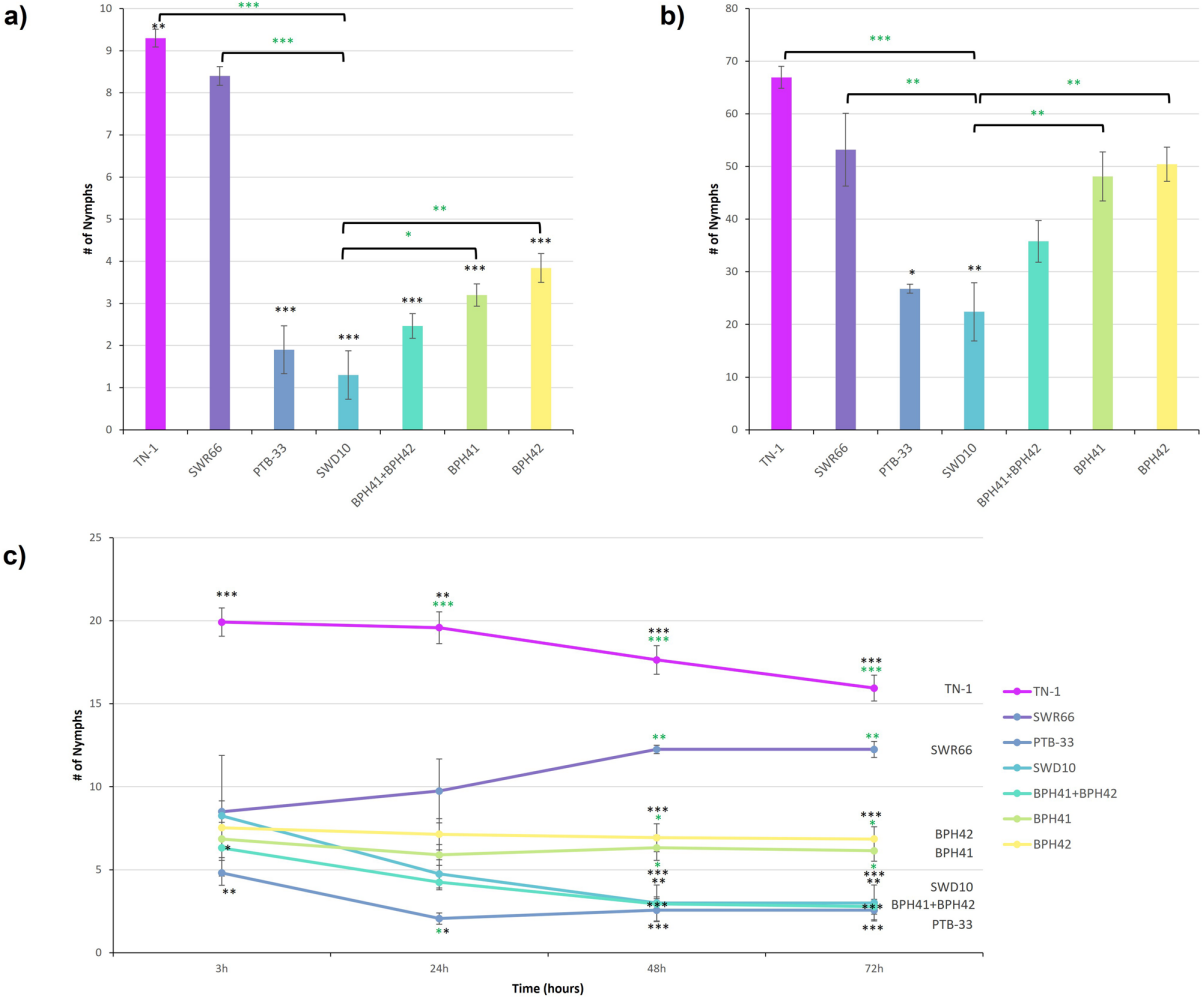
Antibiosis and antixenosis resistance mechanism test results. All genotypes are lines containing the respective resistant allele of the indicated QTLs in the SWR66 background except parental and control lines. (**a**) Results for nymph survival antibiosis test. The number on the y-axis indicates the mean number of nymphs that survived in the antibiosis nymph survival test 20 days post BPH infestation (mean ± s.e; n = 10). (**b**) Results for population growth antibiosis. The number on the y-axis indicates the mean number of F_1_ nymphs that emerged after 35 days post BPH infestation (mean ± s.e; n = 5). (**c**) Results for antixenosis test. The number on the y-axis indicates the mean number of nymphs that remain on each genotype, counted at 3 h, 24 h, 48 h and 72 h post BPH infestation (mean ± s.e; n = 4). Black “*” indicate genotypes that are significantly different compared to SWR66. Green “*” indicate genotypes that are significantly different compared to SWD10. *p value < 0.05, **p value < 0.01, ***p value < 0.001.

Results from the nymph survival antibiosis test showed that the number of surviving nymphs for lines containing homozygous resistant alleles of BPH41 and BPH42 individually, and BPH41 and BPH42 in combination, were significantly lower when compared to the susceptible parent. However, when compared to SWD10, the introgression lines were significantly less resistant (Fig. 4a). In the population growth antibiosis test, similar results to the nymph survival test were observed (Fig. 4b).

In the antixenosis test, the antixenosis mechanism against BPH infestation gradually takes effect over time and was clearly visible after 48 h. Lines containing homozygous resistant alleles of BPH41 and BPH42 individually showed significantly lower number of BPH on the plants at 48 h post infestation compared to SWR66. However, when compared to the resistant donor, SWD10, the antixenosis effect of the individual QTLs were less effective. The number of nymphs at 48 h post-infestation on the genotype containing both homozygous resistant alleles of BPH41 and BPH42 in combination were comparable to that of SWD10 (Fig. 4c). This observation indicates that resistant alleles of BPH41 and BPH42 provide an additive antixenosis mechanism by deterring the BPH to rest on the rice plant.

**Figure 5:**
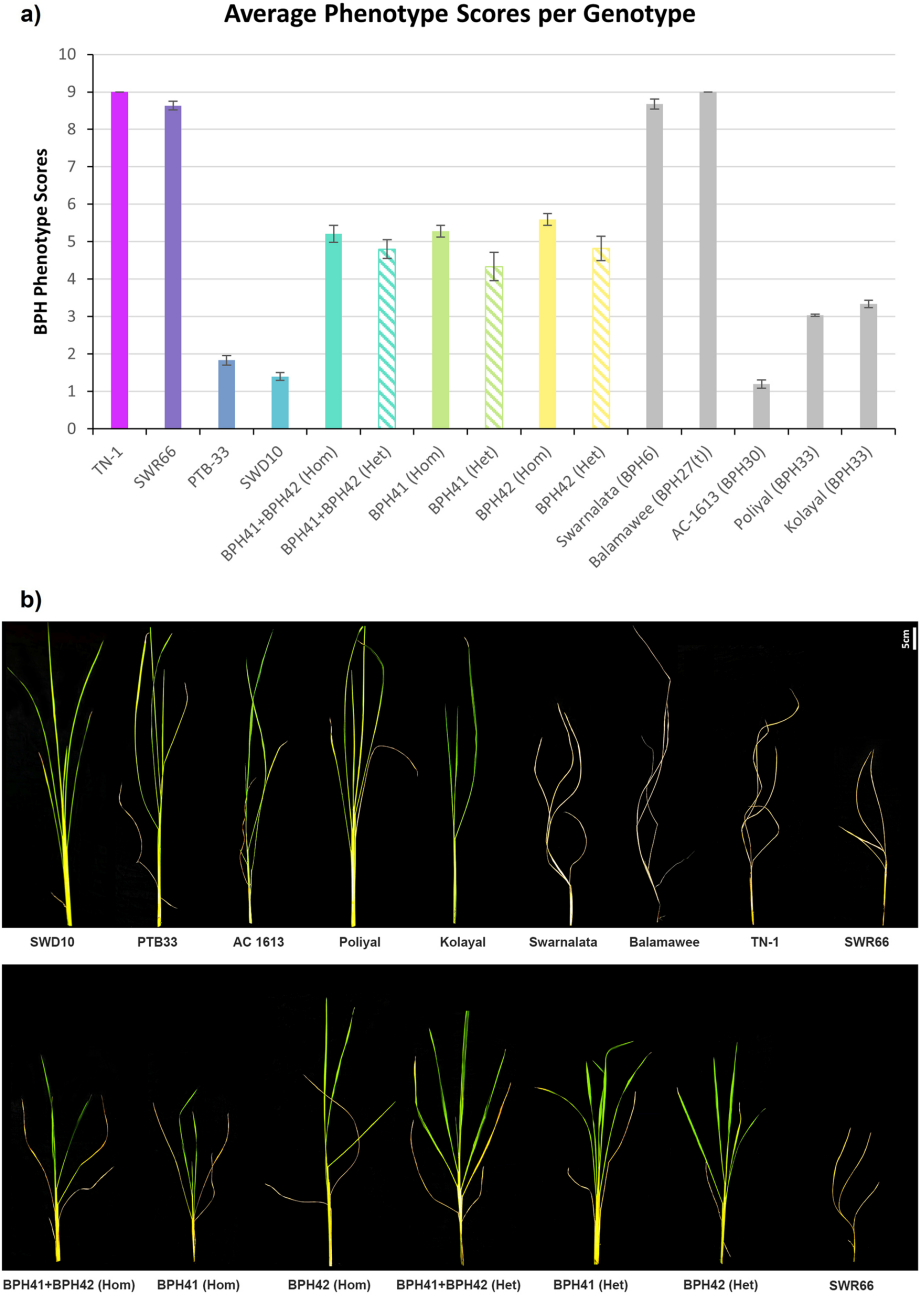
BPH resistance scores for genotypes used in this study. (**a**) Solid and striped bars indicate homozygous and heterozygous genotypes, respectively. Coloured bars are parent genotypes and genotypes carrying the homozygous resistant alleles of the BPH41 and BPH42 that have been used throughout the study. Grey bars are genotypes that were previously reported containing QTLs in the BPH41 and BPH42 region. Values on the y-axis are mean resistance scores (mean ± s.e; n = 60). (**b**) Images representing average BPH resistance phenotype for all genotypes used in this study.

Furthermore, phenotyping results also indicate that the resistant alleles of BPH41 and BPH42 are both dominant QTLs, because the heterozygous genotypes of BPH41 and BPH42 individually exhibited resistance scores that were comparable to their homozygous resistant counterparts (Fig. 5a,b). SWD10 was also significantly more resistant than plants containing resistant alleles of BPH41, BPH42 and BPH41 + BPH42.

### Phenotype and genotype comparison of published QTLs

To identify if the newly mapped QTLs are the same as previously published QTLs, the physical positions of BPH41 and BPH42 were compared to the genetic positions of previously mapped QTLs. The sequences of markers delimiting the regions from previously mapped QTLs on chromosome 4 were used to BLAST against the R498 genome and compared with the location of BPH41 and BPH42 using the same reference genome. The mapped region of BPH41 coincided with BPH30, which was identified from AC-1613^20^ and BPH33, from Kolayal and Poliyal^21^. BPH42 overlaps with the fine mapped region of BPH6, from Swarnalata^19^, BPH27(t), from Balamawee^28^ and BPH34, from IRGC 104646^27^. BPH3, BPH27 and bph18(t) are also located on chromosome 4, however these gene/QTLs are not within the same mapped location as BPH41 nor BPH42, therefore, genotypes carrying these QTLs were not used in this experiment^39–41^. A map depicting the location of all the above-mentioned genes and/or QTLs is presented in Figure S4.

All the genotypes mentioned above except for IRGC 104646 (which we were unable to obtain) were phenotyped for BPH resistance. All lines with previously mapped QTLs were either more resistant or more susceptible compared to the lines carrying resistant alleles of BPH41 and BPH42 individually. BPH30 from AC-1613 and BPH33 from Kolayal and Poliyal were more resistant compared to the homozygous resistant allele of BPH41 alone, with average resistance scores of 1.20, 3.03 and 3.34, respectively. The homozygous resistant allele of BPH41 has an average resistance score of 5.28. BPH6 from Swarnalata and BPH27(t) from Balamawee were susceptible compared to BPH42 alone, with average resistance scores of 8.67 and 9.00, respectively. The homozygous resistant allele BPH42 has an average resistance score of 5.59 (Fig. 5a).

## Discussion

The rice chromosome 4 harbours 12 reported QTLs, making up three major clusters of BPH QTLs. Two of these clusters are located on the short arm and one cluster on the long arm of chromosome 4. Only two BPH resistance genes within these QTL clusters have been cloned, namely, BPH3^14^ and BPH6^19^. The physical locations of BPH41 and BPH42 coincided with two major QTL clusters harbouring five reported QTLs and/or genes. These QTLs and/or genes are BPH6, BPH27, BPH30, BPH33, and BPH34. BPH resistance scores of the genotypes carrying the resistant alleles of these QTLs compared to lines containing homozygous resistant allele of BPH41 and BPH42 individually are different. Therefore, preliminary observations indicate that BPH41 is either a new QTL or contains genes that are allelic to the previously published BPH30 or BPH33. On the other hand, the homozygous resistant allele of BPH42 is phenotypically different when compared to BPH6 and BPH27, but we are unable to conclude that the resistant allele BPH42 is different from BPH34 due to the unavailability of the IRGC 104646 genotype. A more comprehensive confirmation could be carried out by comparing the phenotypic reactions for all QTLs against the same genetic background, however more time is needed to generate these genotypes.

The resistant alleles of BPH41 and BPH42 also contribute equally and independently to provide resistance towards BPH infestation and are dominant QTLs. Despite similar resistance scores observed for each QTL individually, some differences were detected when the reaction towards different resistance mechanisms were considered. Results from the antixenosis and antibiosis tests indicated that both mechanisms contributed similar resistance levels towards BPH when the resistant alleles of BPH41 and BPH42 were considered individually. However, additive effects of resistant alleles of BPH41 and BPH42 were observed for the antixenosis mechanism. The antixenosis effect of resistant BPH41 allele in combination with resistant BPH42 allele is comparable to the original donor parent, SWD10. We postulate that the additive antixenosis effect provides additional protection to the plant and it may be the reason for two similar effect BPH QTLs occurring in the same genotype.

Our attempt to delimit the mapped region of BPH41 and BPH42 by breaking the linkage between the QTLs and identifying recombinant genotypes resulted in screening over 14,368 plants. Through this process, we were able to delimit the QTLs to approximately 540,000 bp and 1,280,000 bp for BPH41 and BPH42, respectively. To further narrow down the potential gene candidates in these QTLs, we looked at the number of annotated genes within the delimited regions. There are 47 and 162 genes annotated genes in BPH41 and BPH42, respectively. Based on the number of annotated genes observed, we could not effectively shortlist the gene candidates. Therefore, we attempted to use the RNA-seq method to further shortlist gene candidates in the QTLs.

By comparing expression profiles generated using RNA-seq for the resistant and susceptible lines, we discovered that, SWD10 may potentially be expressing genes that contribute to BPH resistance before BPH infestation and by being unresponsive to BPH feeding. This observation contrasts with the DEG profile of SWR66 over time. Subsequently, seven candidate genes that may be involved in BPH resistance contributed by BPH41 and BPH42 were shortlisted by comparing and matching the expression profiles of the identified DEGs against the expression profile of the resistant and susceptible parent at 0 h post BPH infestation. Since the genes that were shortlisted are expressed before BPH infestation, we postulate that the gene candidates for BPH41 and BPH42 may be part of the plant innate immunity.

For each QTL, an LRR domain containing gene was identified as a prime gene candidate. The LRR domain containing genes are known to be involved in disease resistance. Many cloned BPH resistance genes, such as BPH1/9, BPH14 and BPH29 are LRR domain containing genes^13,16,18,19,42^. Another gene of interest is the G-patch domain containing gene identified in the region of BPH41. The G-patch domain is mostly associated with RNA processing and is suggested to be a RNA-binding domain mediating RNA–protein interaction^43^. In *Arabidopsis thaliana*, a known G-patch domain containing protein, MOS2, was reported to be involved in the innate immunity against a virulent bacterial pathogen by being critical component in the LRR mediated resistance pathway^44^. To further confirm the findings and to understand the BPH resistance mechanisms contributed by BPH41 and BPH42 in rice, detailed functional studies on the shortlisted genes are required.

The resistant donor, SWD10, also consistently demonstrated superior resistance towards BPH when compared to the lines containing BPH41 and BPH42 alone or in combination. Based on our findings, we strongly postulate that SWD10 contains multiple BPH resistance genes that may have a smaller but significant effect on BPH resistance. To identify these QTLs, a larger mapping population or other methods with higher marker density such as bulked segregant analysis (BSA) may be needed.

Comparing the BPH resistance identified from previous studies with BPH41 and BPH42, BPH6 and BPH27(t) were found to be susceptible in our study. The BPH population that was used to phenotype the plants in the original QTL mapping study was not the same as the ones used in this study. BPH6, BPH27(t), BPH30 and BPH33 were all mapped using BPH populations collected from the rice fields of China which are broadly classified as biotype 1^20,21,28,45^. Identification of some of these resistant lines were also first discovered in the 1970s using BPH biotypes of 1 2 and 3 but not the South Asia biotype, biotype 4^46^. Due to the different sources of BPH populations, this may be the reason that the previously published QTLs may be ineffective against the BPH populations collected from India.

Furthermore, application of BPH biotypes in research is now almost obsolete due to quick resistance developed against BPH resistance genes and also the confusion of naming biotypes^47^. BPH populations have quickly evolved over time and the discovered BPH resistance QTLs may have been rendered ineffective against the BPH populations such as reported for the BPH resistance QTLs of BPH1 and BPH2^29^.

Therefore, our findings from this study are particularly applicable for breeding BPH resistance rice especially in South Asia. The discovery of BPH41 and BPH42 from SWD10 will greatly enhance the selections of available BPH resistance genes for breeders. The materials generated from this study can be directly applied in rice breeding programs. These materials can be used to better understand BPH resistance genes and how they interact with each other in cultivated rice. Using the information generated from these materials, breeders will be able to choose between different combinations of BPH resistance genes to include in their breeding programs. In summary, the newly identified QTLs and information gathered from this study have expanded the currently available toolkit for breeding better BPH resistant rice varieties.

## Materials and methods

### Plant materials

All *O. sativa* lines of SWR66, SWD10 and its progenies, fixed lines of PTB33 (IRGC 19325), TN-1 (IRGC 38845), AC-1613 (IRGC 10638), Kolayal (IRGC 36295), Poliyal (IRGC 36352), Swarnalata (IRGC 33964) and Balamawee (IRGC 40777) were kindly provided by SeedWorks International Pvt. Ltd. The 232 individuals used for the F_2_ mapping population were created by crossing the susceptible parent, SWR66, with the resistant parent, SWD10. Recombinant lines containing different recombination fragments of the QTLs were selected from the initial F_2_ population. These lines were further selfed or backcrossed into SWR66 to generate advanced generations of F_3_ to F_6_, BC_1_F_1_ to BC_1_F_4_ and BC_2_F_1_ to BC_2_F_4_ individuals. A total of 14,368 recombinant lines were screened to delimit the QTLs.

### Insect materials

BPH were collected from the rice fields in Rajahmundry, Andhra Pradesh State, India and were maintained by rearing in BPH cages. Adult gravid female hoppers were collected and transferred onto pre-cleaned potted plants of TN-1 inside oviposition cages. Every 2 years, the BPH were mixed with newly caught BPH from the rice fields in Rajahmundry, India to maintain virulence.

### BPH resistance phenotyping

Phenotyping was done at the seedling stage in the greenhouse following the International Rice Research Institute (IRRI) standardized procedures described earlier^37,38^. Within a tray, each entry of rice seedlings was grown in rows of 30 seeds with 5 cm spacing between each row in standing water. The first and last rows were planted with the susceptible check (TN-1) and the middle row was planted with the resistant check (PTB33). During the 6th day after sowing the seeds, each row will be thinned to only contain 20–30 plants per row. 2nd and 3rd instar BPH nymphs were used to infect 10–12-day-old rice seedlings. Each seedling was infested with a density of 5–7 nymphs. The seedlings were then covered with nylon mesh after infestation. Resistance scores were recorded when approximately 100% of the susceptible checks were killed. Each plant was rated using the standard evaluation system for rice^38^. Individual seedlings were scored for resistance, and the average resistance score was taken as the resistance score of the F_2_ plant.

### Antixenosis and antibiosis tests

The antixenosis and antiboisis tests were carried out using a tray setup previously described^37^ with modifications. Genotypes used for the antixenosis and antibiosis test were (1) susceptible parent SWR66, (2) resistant parent SWD10, (3) individuals containing the homozygous resistant allele of QTL1, and (4) individuals containing the homozygous resistant allele of QTL2, (5) individuals containing both the homozygous resistant alleles of QTL1 and QTL2, (6) resistant check PTB33 and (7) susceptible check TN-1.

For the antixenosis mechanism test, a tray containing genotypes mentioned above was prepared for each replicate. Each genotype is referred to as a hill. Approximately ten BPH nymphs were released on each hill. The number of nymphs on each hill were counted at 3 h, 24 h, 48 h and 72 h post infestation. The experiment was conducted in four replicates.

Two antibiosis tests were conducted, (1) the nymph survival test and (2) the population growth test. The nymph survival test was carried out by releasing 10 nymphs in each pot, each containing one plant. The number of surviving nymphs were calculated 20 days post infestation. The experiment was conducted in ten replicates.

The population growth test was conducted by placing two pairs of adult BPH in each pot containing three plants. Each pot only contained one genotype. The number of F_1_ nymphs that emerged were counted 25 days post insect infestation. The experiment was conducted in five replicates.

### SNP genotyping

DNA was extracted and purified using the sbeadex purification kit and recommendations by LGC Genomics, UK. SNP genotyping was performed using the KASP genotyping chemistry on purified DNA. All SNP markers used in the experiments were developed by Straits Biotech Pte. Ltd. The resulting plate reader results were imported into the Kraken software for scoring and visualization, Biosearch Technologies, LGC.

### Genetic map construction and QTL mapping

Statistical analysis for both genetic map construction and QTL mapping was carried out using the R/qtl package. First the genetic map of polymorphic markers for the population SWR66 × SWD10 was determined using the onemap package^48^. The genetic distances between individual markers based on the genotypes of the F_2_ population was calculated based on the Kosambi mapping function. A mapmaker file containing both the genotypes and the average resistance score of each individual F_2_ was used as input for the R/qtl package.

QTL mapping was done using the R/qtl package^49^. Three QTL mapping algorithms, expectation–maximization (EM) algorithm, Haley Knott Regression (HK) and the multiple imputation algorithm (imp), were utilized and compared. Permutation test with 1000 iterations were conducted for each algorithm to estimate the threshold LOD value and the maximum LOD threshold among all three algorithms was used as the LOD threshold. Furthermore, effect and interaction of each detected QTL was estimated using the full model of $y\sim BPH41+BPH42+BPH41:BPH42$.

### Whole genome resequencing of parental lines

Whole genome re-sequencing was conducted for both SWR66 and SWD10. DNA extraction of fresh young leaf tissues from a 2-week-old plant was done using the DNeasy Plant Mini Kit (Qiagen, Germany). The library prep and subsequent whole genome re-sequencing experiment was outsourced to Axil Scientific Pte. Ltd. Samples were sequenced with a sequencing depth of 30× using the Hiseq 4000. The resulting fastq files were aligned to the reference genome of R498^50^. The SNP calling criteria was based on Q30 greater than 90% and read depth of at least 100×. The resulting variant call format (VCF) output file was used to filter out SNPs that are located within the QTL regions.

### RNA-seq

four genotypes were utilized for this experiment: (1) SWR66, (2) SWD10, (3) BC_2_F_2_ individuals containing the homozygous resistant allele of BPH41 and, (4) BC_2_F_2_ individuals containing the homozygous resistant allele of BPH42. For each genotype, a 2-week-old seedling was planted in a pot per replicate. The seedling was infested with ten 2nd to 3rd instar nymphs and each pot was covered with Mylar sheets. Approximately 2 cm of rice seedling stems above the water was collected at the 0 h and 24 h post infestation. The experiments were designed to start at different times but ended at the same time. The experiment was conducted with three biological replicates in a greenhouse which was maintained at a temperature between the ranges of 25–30 °C and relative humidity of 70–80%.

RNA samples were submitted to the Genome Institute of Singapore (GIS) for library preparation, RNA-seq and subsequent bioinformatic analysis for differential gene expression (DEG) studies. The resulting fastq files were aligned to the reference genome of *O. sativa* ssp. *indica* ShuHui R498^50^ and the corresponding version 3 gene annotation file. STARR aligner was used to map RNA-seq sequences to the reference rice genome^51^. Subsequent analysis for DEG was performed with DESeq2^52^. Genes with total read counts of less than 20 across all samples were removed from the DESeq2 analysis.

Additionally, gene ontology (GO) term analysis for DEG was conducted using the TopGO R package^53^. DEGs between time points for each genotype were filtered based on the following criteria: p value less than 0.001 and absolute log_2_ fold change of greater than 2. Filtered DEGs were used for GO analysis based on GO terms downloaded from the *O. sativa* ssp. *indica* R498 v3 annotation file^50^.

KEGG pathway analysis was also conducted using the KOBAS software^54^. The fasta sequences of DEG with the same criteria used in the GO term analysis were obtained using seqtk (https://github.com/lh3/seqtk). Subsequently, the resulting fasta file was used as input against the *O. sativa* ssp. *japonica* RefSeq database to generate significant KEGG pathways^55–57^.

### qRT-PCR

Three biological replicates per genotype were obtained using three separate individuals. Each genotype was subjected to a time course experiment identical to the RNA-seq experiment. qRT-PCR was carried out using three technical replicates per genotype for each timepoint. cDNA was synthesized from RNA according to the recommended protocol for Maxima First Strand cDNA Synthesis Kit for qRT-PCR, with dsDNase (Thermo Scientific, United States). The qRT-PCR reaction was prepared according to the recommended KAPA SYBR Fast qPCR Master Mix (Roche, Switzerland). The gene, LOC_Os03g13170, encoding for ubiquitin was used as a housekeeping gene^58^. Relative quantity of RNA for each gene was calculated using SWR66 at 0 h as the reference. Student’s *t* test was used to calculate significance of relative RNA quantities between genotypes.

## Supplementary Information

Supplementary Information 1.

Supplementary Information 2.

Supplementary Information 3.

Supplementary Information 4.

Supplementary Information 5.

Supplementary Information 6.

## Data Availability

Authors can confirm that all relevant data are included in the article and/or its supplementary materials.
