# Supplementary material for: Identification of two QTLs, BPH41 and BPH42, and their respective gene candidates for brown planthopper resistance in rice
Source: Sci Rep. 2022 Nov 2;12:18538. doi: 10.1038/s41598-022-21973-z (PMC9630283; doi:10.1038/s41598-022-21973-z)

Identification of two QTLs, BPH41 and BPH42, and their respective gene candidates for brown planthopper resistance in rice

Han Qi Tan^1,2^, Sreekanth Palyam^3^, Jagadeesha Gouda^3^, Prakash P. Kumar^1^, Santhosh Kumar Chellian ^2^

^1^ Department of Biological Sciences, National University of Singapore, Singapore.

^2^ Straits Biotech Pte. Ltd., Singapore.

^3^ SeedWorks International Pvt. Ltd., Hyderabad, India.

* Corresponding author: Santhosh Kumar Chellian (santhosh.chellian@straitsbiotech.com)

Short Legends for Supplementary Information

**Figure S1.** BPH resistance scores distribution of F_2_ mapping population

**Figure S2.** Venn diagram comparing DEG for SWD10 and SWR66 at time 6 h and 24 h

**Figure S3.** qRT-PCR confirmation of RNA-seq Results

**Figure S4.** Map of identified QTLs relative to BPH41 and BPH42 on chromosome 4

**Table S1.** qRT-PCR primers used in this study

**Table S2.** SNP and flanking sequences used in this study

**Data S1.** Genotypes, phenotypes, markers, genetic and physical positions of markers used in F_2_ mapping population

**Data S2.** List of genes located within the delimited region of BPH41

**Data S3.** List of genes located within the delimited region of BPH42

**Data S4.** Metadata of RNA-seq samples

**Data S5.** Gene Count table for RNA-seq

Supplementary Figure Legends

**Figure S1.** BPH resistance score distribution of F_2_ mapping population. BPH resistance scores of parental lines, SWD10 and SWR66, are indicated with red arrows. 0-1 is highly resistant, 1-3 is resistant, 3-5 is moderately resistant, 5-7 is moderately susceptible and 7-9 is susceptible and 9 is highly susceptible. BPH resistance scores were calculated based on the mean of 90 F_3_ individual resistance scores per F_2_ individual and total number of plants that fall into each category was plotted.

**Figure S2.** qRT-PCR confirmation of RNA-Seq Results. Expression for five of the seven shortlisted genes were confirmed using qRT-PCR. All five genes were verified using three biological replicates and three technical replicates. a-b) Shortlisted genes located in BPH41. These genes are OsR498G0407469100.01, OsR498G0407476800.01, respectively (mean ± s.e; n=3). c-e) Shortlisted genes located in BPH42. These genes are OsR498G0408591800.01, OsR498G0408592800.01, and OsR498G0408592900.01, respectively (mean ± s.e; n=3). Within each plot, gene expression levels of four genotypes at 0 h, and 24 h are indicated by four bars on the left and right, respectively. Gene expression levels at 0 h confirm the DEG results from the RNA-Seq analysis. Gene expression levels at 24 h were used as further confirmation of the RNA-Seq data. “*” indicate genotypes with gene expression levels that are significantly different compared to SWR66 for each timepoint. * p-value<0.05, ** p-value <0.01, *** p-value <0.001.

**Figure S3.** Venn diagram comparing DEG for SWD10 and SWR66 at time 6 h and 24 h. Venn diagram illustrating the number of differentially expressed genes comparing SWR66 and SWD10 with the same genotypes before BPH infestation at 0 h. Cut-off values, p-value < 0.001 and absolute log_2_ fold change >2 were used to identify significant DEGs. a) DEG of SWR66 and SWD10 at 6 h. b) DEG of SWR66 and SWD10 at 24 h.

**Figure S4.** Map of identified QTLs relative to BPH41 and BPH42 on chromosome 4. SNP markers used in this study are indicated on the right side of the chromosome cartoon. Markers in red fonts are markers closest to the QTL peaks identified in BPH41 and BPH42. The genetic map is indicated with a scale of every 5 cM on the left of the chromosome cartoon. QTLs indicated with solid red bars and red fonts are BPH41 and BPH42 identified in the current study. Solid green bars with green fonts indicate BPH genes that were cloned prior to this study. Black striped bars indicate the relative genetic positions of QTLs on chromosome 4 identified prior to this study.

**Table S1. qRT-PCR primers used in this study**

| **Primer ID** | **Sequence 5'-3'** |
| --- | --- |
| LOC_Os03g13170_Ubi_F | GTATCATCGAGCCGTCGCTTC |
| LOC_Os03g13170_Ubi_R | CATAGCATTTGCGGCAGATCA |
| OsR498G0407469100.01_2F | CCACGGTGTTTGCACGATAC |
| OsR498G0407469100.01_2R | TGCCCAATGGTGAGCTTGAT |
| OsR498G0407476800.01_2F | GCAGCTTTCGGATTACCGAC |
| OsR498G0407476800.01_2R | TATAGCGGCATCACTCAGCC |
| OsR498G0408591800.01_1F | GCCATAGATGAATATTTCAAACCC |
| OsR498G0408591800.01_1R | TTTGCGTTTGGGCGTCTTTA |
| OsR498G0408592800.01_2F | TGATGGAGATGGCGCACAC |
| OsR498G0408592800.01_2R | ACGGAGATGTAGGAGCAGGT |
| OsR498G0408592900.01_1F | GCAGCTTTGGTCTGCCTTCT |
| OsR498G0408592900.01_1R | ATCTCAGAGTAACGCTCCCTT |

**Table S2. SNP and flanking sequences used in this study**

| **Marker ID** | **SNP Sequence** |
| --- | --- |
| SWRm_01617 | TGTATTCCAAGGTAAACCCAGCAACTCTTTGCAAAGGAGCAGGAGAAAAA[A/G]AGATGGGTCGTGTCTTCAARGGCTGCATCACTGCACATTACACATTCTGG |
| SWRm_01636 | TACTCCTACGTGGCTATGGTYGAGAGAGCATGGACATCATCAGTGAGCTT[A/C]AGCTATGGCGCGCTCGTCGGCTTGCTTCCATTCCATTTTGACGTCCTAAT |
| SWRm_01522 | TGTTGAGGACTTGATACTGGATTGGCATGTTCTGTTGTAACGACCACAAC[T/C]AATATGTATATTATATTTAGTTCACCAACTATACATATATGTTGGAGTCT |
| SWRm_01695 | TCTATCATTGGGTTATCATTAGGCTAGAGGGCCGCTCCACCAACTCTATC[C/T]AACTGCATGGTCACTCCTATCACGTCATTCTTGCTATAGTGCGATAATAT |
| SWRm_00328 | TCGCCGCCGCCGCTTCGCTCTCATCTCTCCCCGTCTCCGTCTCCACCACCATGGATTGAT[A/G]CCTCAGAAAGTCTTCGGTCTTCGTCGGAGTCACCAATCACATGGCGATTGCGAGCACGCA |

**Figure S1.** BPH resistance score distribution of F_2_ mapping population.


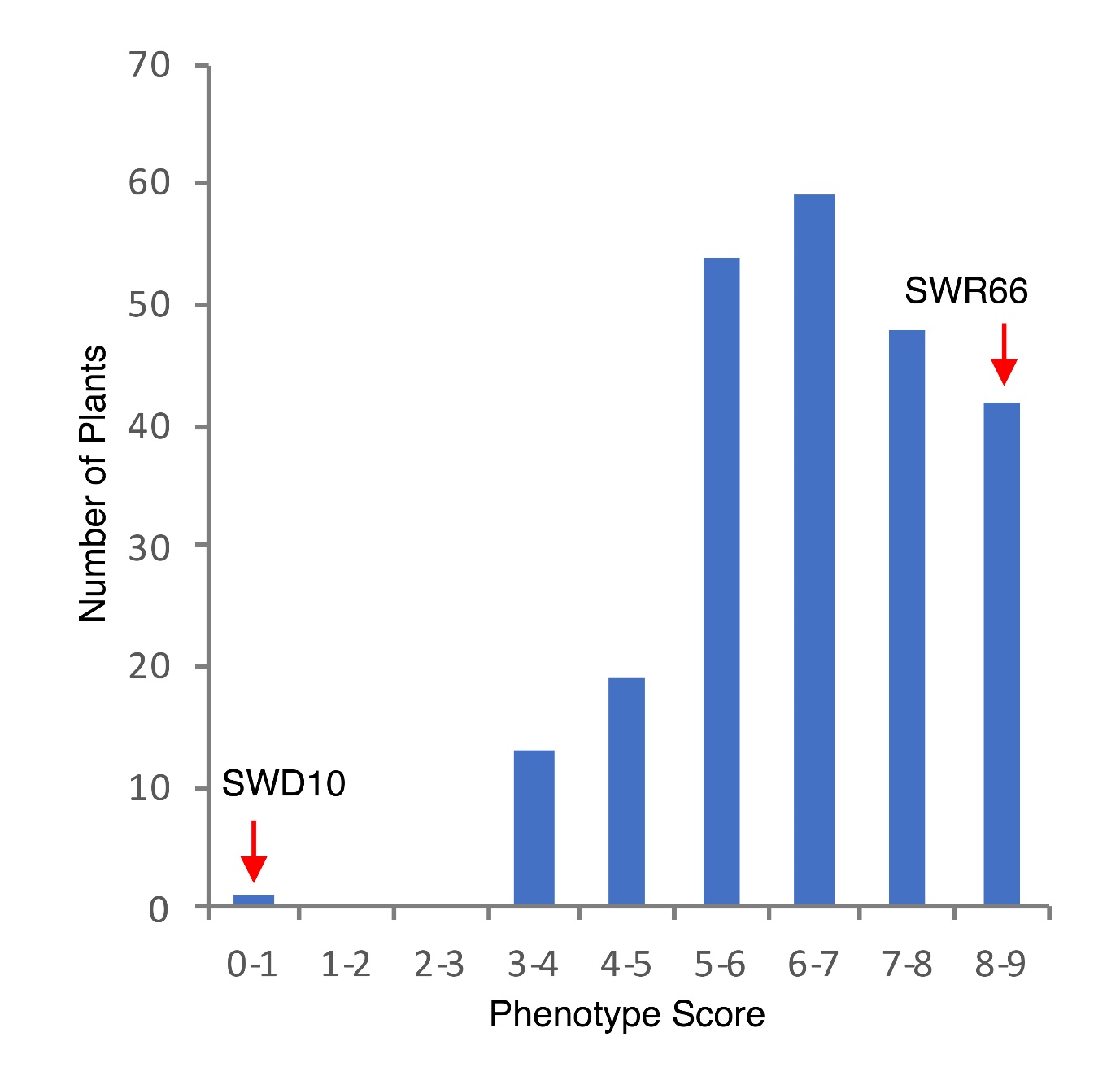


**Figure S2.** qRT-PCR confirmation of RNA-Seq Results.


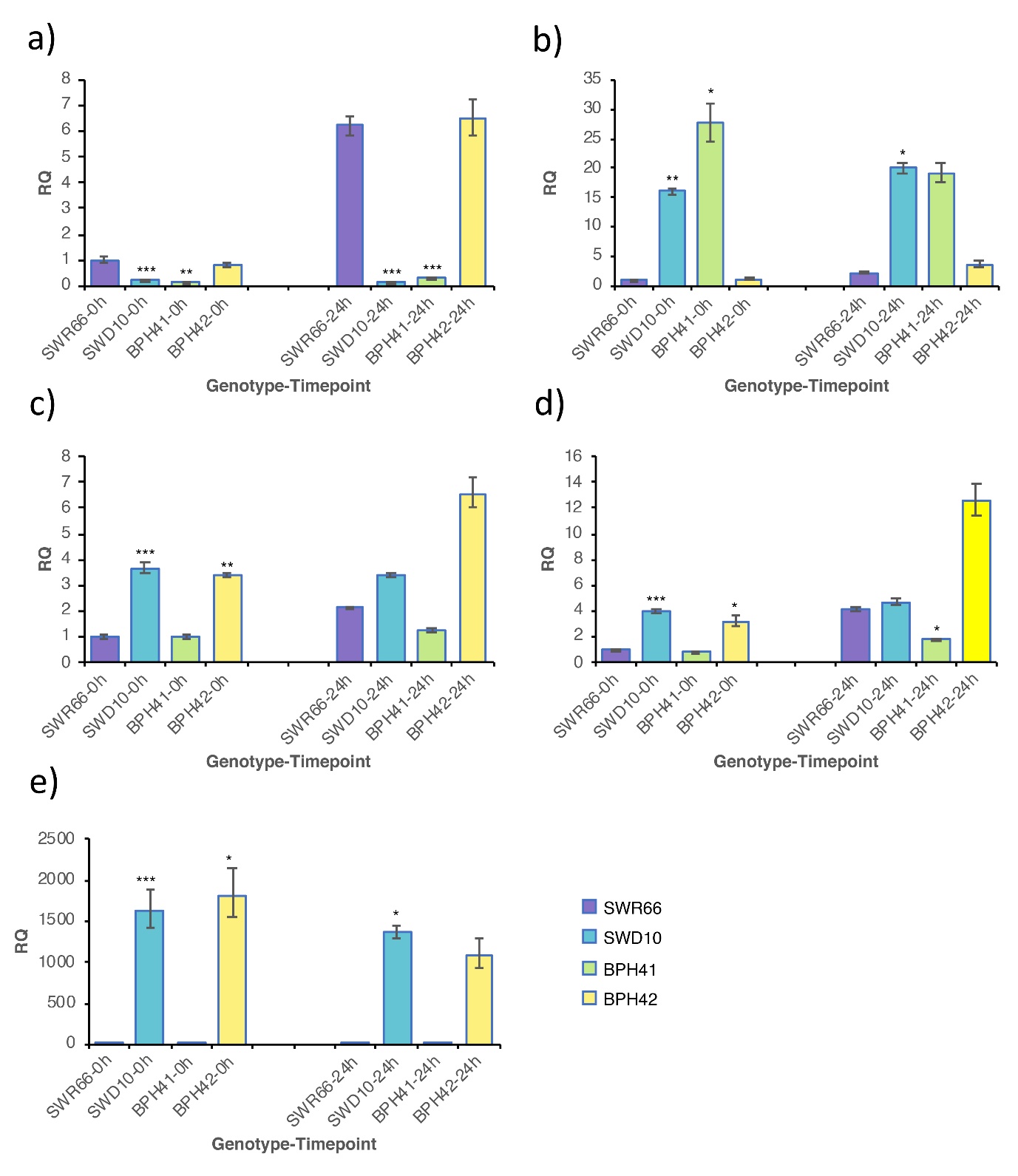


**Figure S3.** Venn diagram comparing DEG for SWD10 and SWR66 at time 6 h and 24 h.


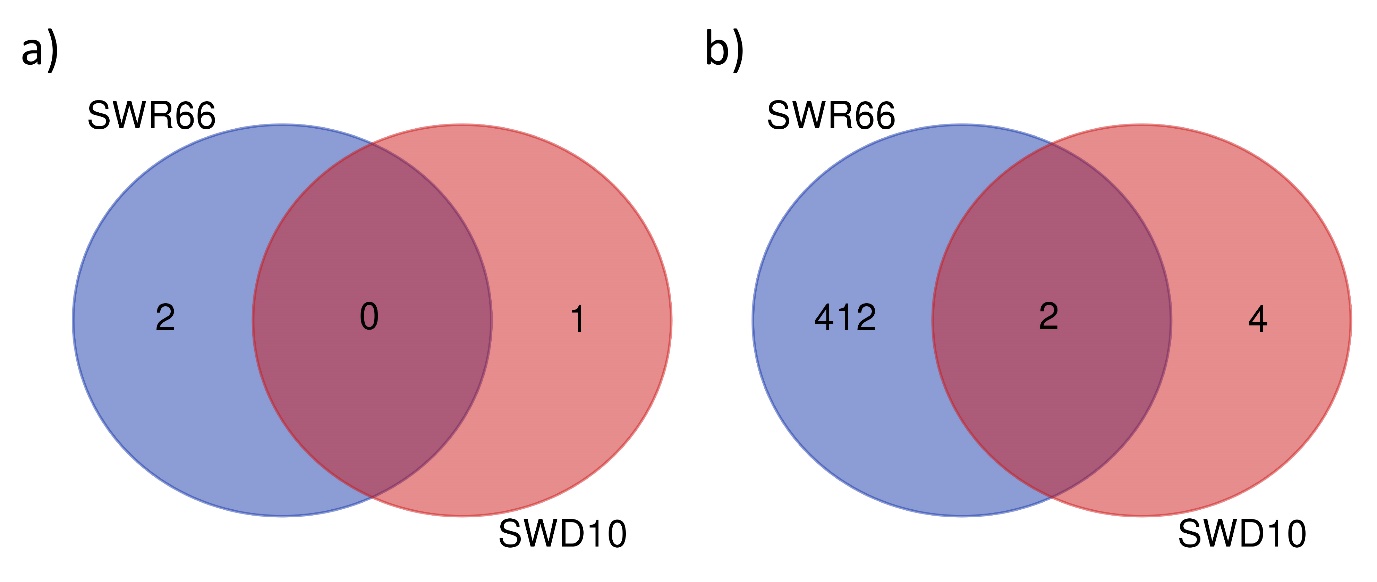


**Figure S4.** Map of identified QTLs relative to BPH41 and BPH42 on chromosome 4.


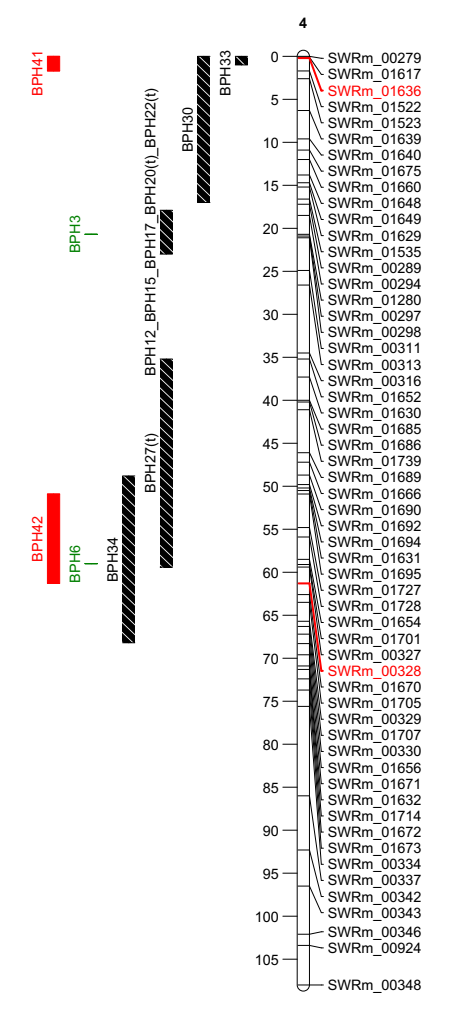

Supplement: Supplementary file 6 — Supplementary Information 6. [file 41598_2022_21973_MOESM6_ESM.docx]
